# Improving the Consent Process for Superficial Abscesses Through Pre-printed Consent Forms

**DOI:** 10.7759/cureus.73421

**Published:** 2024-11-11

**Authors:** Sima Patel, Ceri Gillett

**Affiliations:** 1 Surgery, New Cross Hospital, Wolverhampton, GBR

**Keywords:** consent form, general surgery, patient consent, quality improvement project (qip), superficial abscess

## Abstract

Introduction

Informed consent is essential to ensure that patients are appropriately educated about proposed procedures, including associated risks and potential benefits, to make a valid decision. Incision and drainage of an abscess is a common procedure performed by various healthcare professionals. Inconsistent practices in the consent process can lead to misunderstandings among the patient and have financial and legal complications for both the clinician and the hospital. This study aims to improve the consent process for the incision and drainage of a superficial abscess via the implementation of pre-printed consent forms.

Method

We conducted a retrospective, single-centre study to evaluate existing consent forms, found in patients' notes, for the incision and drainage of superficial abscesses. The goal was to assess these forms for standardisation, ultimately developing a pre-printed consent form suitable for use by a diverse range of healthcare professionals.

Results

This study revealed significant inconsistencies in consent documentation. While 20 out of 22 (91%) consent forms included infection as a documented risk, only 11 out of 22 (50%) mentioned COVID-19 as a potential risk. The study found that 22 of our 22 (100%) consent forms were documented in black ink. Providing patients with copies of the consent forms can enhance their understanding by allowing them to review the information at home. Eight out of 22 (36%) patients were offered a copy, and 13 out of 22 consent forms (59%) were noted to have abbreviations, both of which may limit understanding and comprehension of the procedure. After implementing a pre-printed consent form, 14 clinicians surveyed (100%) reported that the forms were effective, with 12 out of 14 (86%) indicating they would use these pre-printed forms in their future practice.

Conclusion

The identified variations and inconsistencies in the consent process prompted the creation of a standardised pre-printed consent form. Feedback on this form has been positive, indicating its potential to transform the consent process. The sample size was small, so early results are positive; however, further ongoing work would be required to draw a more definitive conclusion.

## Introduction

Within the United Kingdom (UK), physicians who wish to examine, investigate, or initiate a treatment in any patient who is deemed to have capacity are required to obtain consent unless the situation is deemed an emergency or is required by law, such as in patients with psychiatric disorders [1]. The Department of Health (DOH) and the General Medical Council (GMC) both describe the consent process as a shared decision-making process between the clinician and the patient [2,3].

Having capacity means that an individual can make their own decisions. The Mental Capacity Act 2005 (MCA) aims to empower individuals and provides the legal framework for decision-making when patients may lack the capacity to make part of or all their own decisions. It applies to individuals aged 16 years or older, living in England or Wales, and follows the core principles that everyone is deemed to have capacity until proven otherwise, and individuals must be supported in making their decisions. Furthermore, if a person makes a decision that clinicians deem to be unwise, this does not automatically mean that they do not have the capacity [4]. Importantly, the assessment of capacity is decision-specific and time-specific. For the capacity to be present, the patient must be able to fulfil the following four domains: understand the information given, retain the information, weigh up the risks and benefits, and communicate their decision. If an individual is unable to fulfil all four domains, they are deemed to not have capacity. The clinician then needs to decide whether the lack of capacity is temporary or permanent, and if temporary, a decision needs to be made as to whether the intended treatment can wait until capacity is regained [4].

A patient who is deemed to have capacity and is undergoing an examination, investigation, or treatment should be asked for consent [1]. Consent can be verbal, written, or implied. Gaining informed consent requires the clinician to explain the procedure, the intended benefits, and possible risks associated with the procedure, as well as any reasonable alternatives to the procedure, including the risks and benefits of alternative options. Within general surgery, incision and drainage of an abscess is one of the first procedures a junior doctor will learn. As part of the procedure, the junior doctor must be able to take valid consent. Traditionally, written consent is taken from the patient. In the National Health Service (NHS) within the UK, a version of “The Wales” consent form 1 is used to obtain consent from an individual aged 16 or over who is deemed to have capacity. The use of generic consent forms can lead to multiple issues that may invalidate the consent process, such as illegibility and human error, leading to errors on the consent form or missed risks documented and discussed with the patient.

We carried out a study to assess the feasibility of pre-printed, procedure-specific consent forms to be used when obtaining consent for the incision and drainage of an abscess under general anaesthesia. The aim of the study was to improve documentation of written consent by ensuring completeness of the consent form while improving patient understanding of the procedure as well as the risks and benefits associated with it.

## Materials and methods

A retrospective, single-centre study was conducted at the New Cross Hospital, Wolverhampton, UK, designed to assess the quality of the consent process for the incision and drainage of an abscess. Data was collected from the notes of patients who presented to a district general hospital within the NHS, under the general surgical team, with an abscess that required an incision and drainage to be performed.

All consent forms for any individual aged 16 years and over who was deemed to have the capacity to give consent were reviewed. Data was collected over a four-week period in August 2024. Consent forms were assessed for the parameters shown in Table 1.

**Table 1:** The parameters assessed when assessing abscess incision and drainage consent forms.

Parameters assessed
Written in black ink
Legibility
Any abbreviations used
Was a copy of consent given to the patient
Signed by clinician
Signed by patient
Dated by clinician
Dated by patient

When assessing consent forms, the commonly agreed risks associated with incision and drainage that were considered appropriate to be included were pain, bleeding, inflexion, scarring, deep vein thrombosis, recurrence of abscess, and COVID-19 [5].

Any consent form completed for a child (consent form 2) or an individual not deemed to have capacity (consent form 4) was excluded from the data collection. Any incision and drainage documented as being performed under local anaesthesia were also excluded from the data collection. Results from baseline measurements were presented to the general surgical team, and a proposed pre-printed consent form for the incision and drainage of abscesses was generated.

Formation of pre-printed consent forms

**Figure 1:**
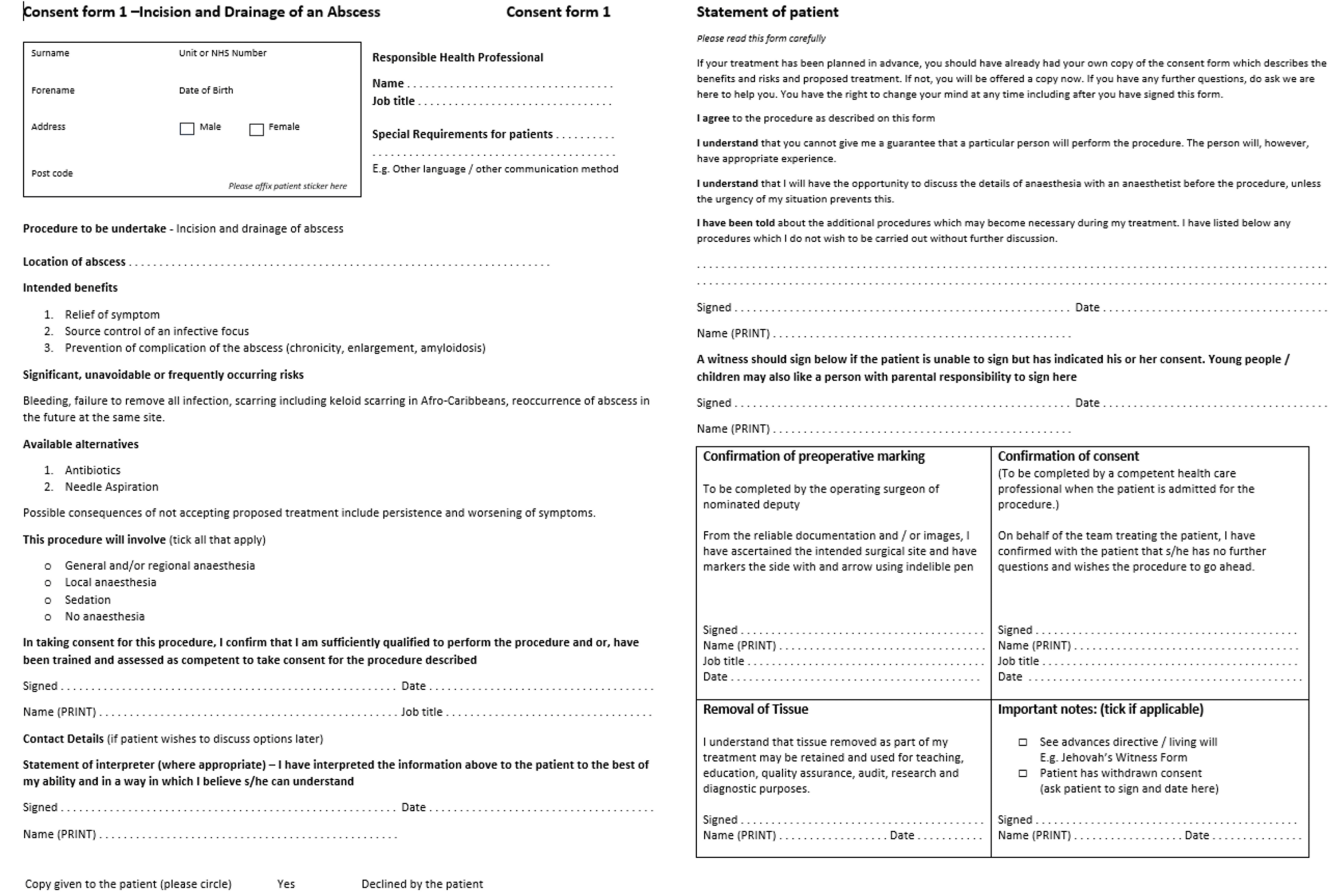
Pre-printed consent forms to be implemented within the general surgical department for the incision and drainage of a cutaneous abscess.

Following a review of baseline measurements along with discussions with two independent colorectal surgeons within the general surgical unit, a pre-printed consent form was developed, taking into account the highlighted issues. The consent forms were designed to be easy to understand by both the patient and the clinician, contain the essential information required for the consent process, and be easy to use. Figure 1 shows the final version of the pre-printed consent forms generated for use.

**Figure 2:**
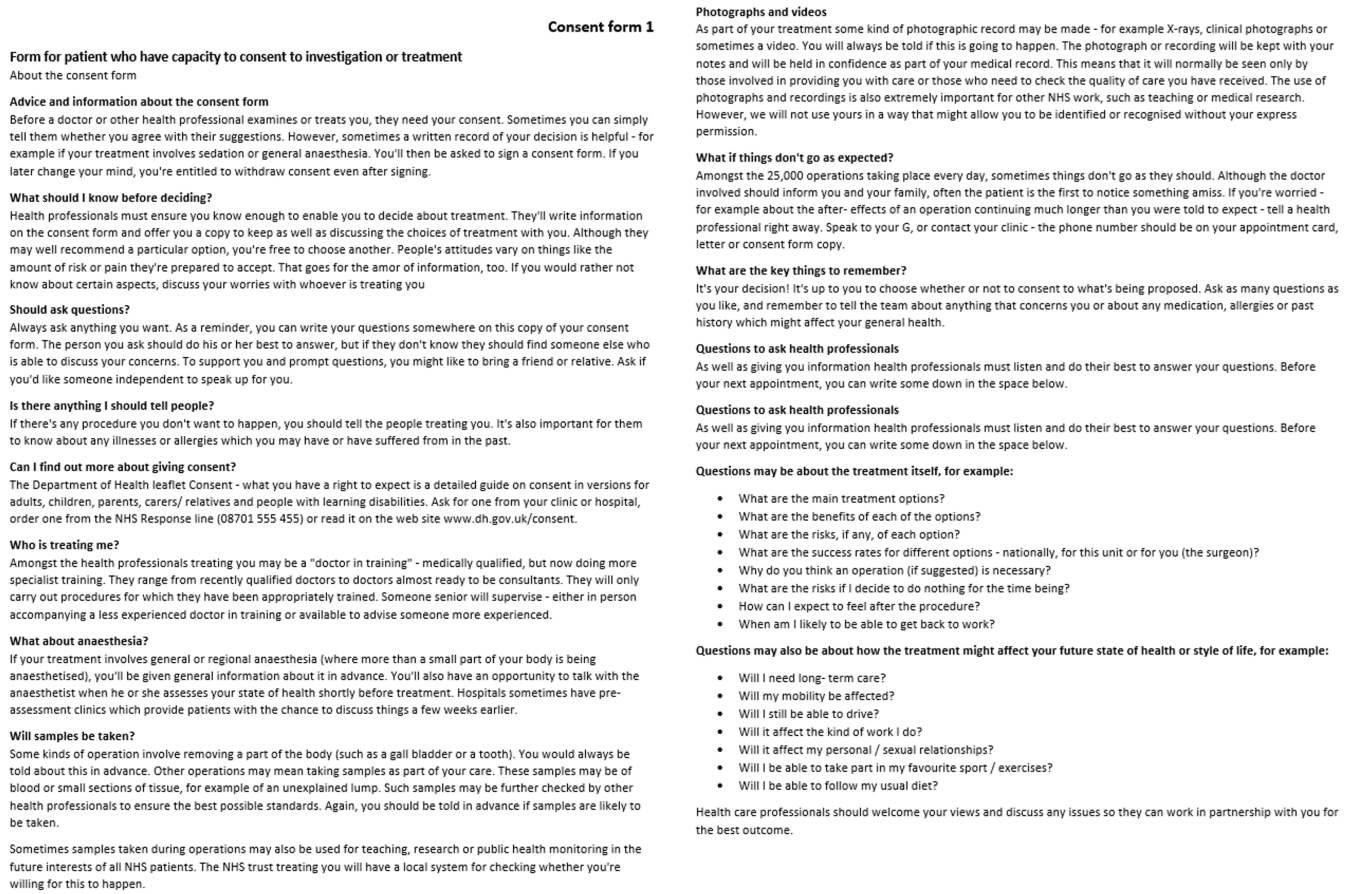
Front cover of pre-printed consent forms to be implemented within the general surgical department for the incision and drainage of a cutaneous abscess, giving information to the patient.

Figure 2 shows the front cover of the pre-printed consent form. This was designed to comply with the requirements for consent forms set out by the Trust in which this study was performed.

Prior to the implementation of the pre-printed consent forms, a talk was given to all junior doctors and clinicians within the general surgical department regarding the prototype of the pre-printed consent forms, how to use them, and when to use them. Junior doctors and clinicians were then surveyed following the talk about the useability of the pre-printed consent forms to assess viability for future practice. The decision to survey junior doctors and clinicians was due to the fact that these individuals were considered most likely to gain patient consent.

Feedback was obtained from junior doctors to assess the usability and overall satisfaction of the consent form prior to implementation.

## Results

Data collected for baseline measurements showed that 38 patients who required an incision and drainage to be performed presented to the general surgical team over four weeks. Of the 38 patients, 16 were excluded from the data collection process. One patient was excluded due to being a child; therefore, parental consent was sought using a version of “The Wales” consent form 2. Nine patients were excluded due to having an incision and drainage performed under local anaesthesia and not having a formal consent form. Four patients were excluded as the patients were deemed to lack capacity; therefore, consent was obtained via a version of “The Wales” consent form 4. Two patients were excluded as the records were incomplete, and therefore, it was unclear how consent was sought.

**Figure 3:**
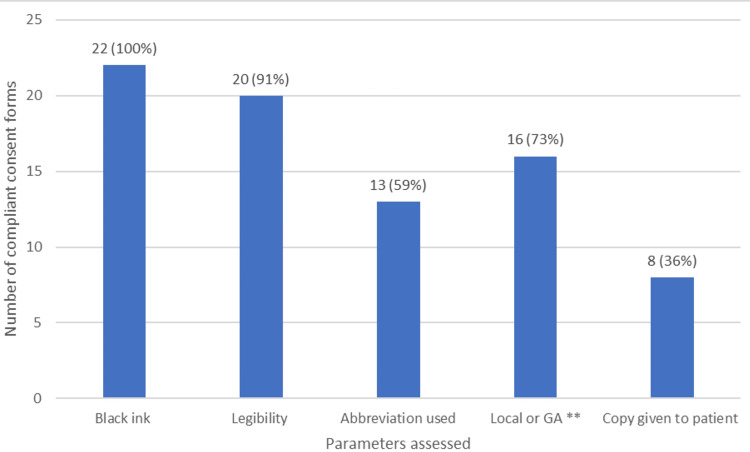
Assessment of parameters that are considered essential for optimal documentation of consent forms, in keeping with the trust policy for documentation. ** Documentation of whether the procedure was done under local or general anaesthesia (GA)

Figure 3 shows the outcome of assessing the quality of documentation using parameters that are considered essential for optimal documentation. The parameters assessed were established using the trust policy for consent at the hospital where this study took place.

Trust policy at the hospital where this study took place required consent forms to be written in black ink. The use of black ink ensures the document is easier to photocopy if required. Legibility was assessed, as illegible writing could affect the patient’s ability to read the consent form, which may hinder the patient in coming to an independent decision about the procedure. Similarly, abbreviations used may be unknown to patients and affect their overall decision-making.

**Figure 4:**
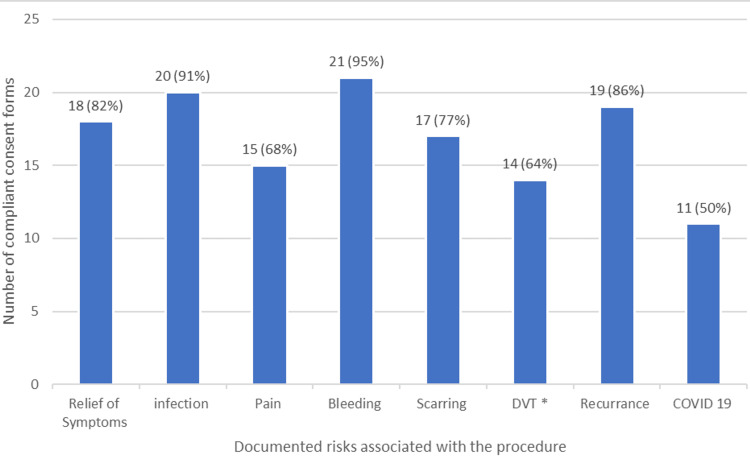
Side effects and risks documented on abscess incision and drainage consent forms. DVT: deep vein thrombosis

Figure 4 shows the different side effects and risks that were documented on consent forms for the incision and drainage of an abscess.

Feedback was obtained from junior doctors to assess the usability and overall satisfaction of the consent form prior to implementation. A feedback survey was undertaken to assess the general consensus on the usability of the pre-printed consent form. Fourteen junior doctors, ranging from foundation year one doctors to registrars, were surveyed, with results in favour of consent from us.

**Figure 5:**
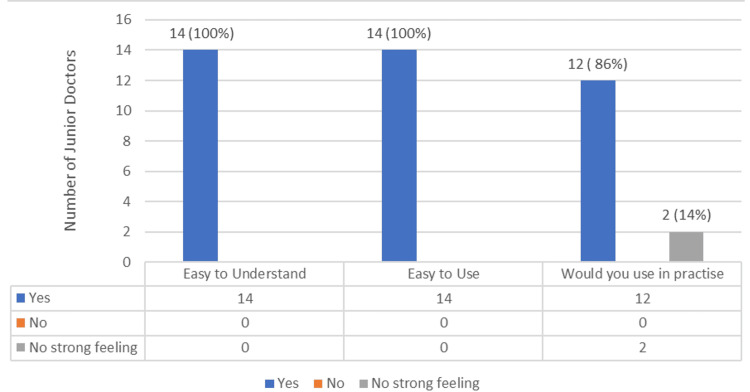
Junior doctor feedback on the pre-printed consent form following introduction.

Overall feedback was positive, with 12 (86%) of those surveyed stating that the pre-printed consent forms would be useful and 14 (100%) stating that the use of pre-printed consent forms would standardise care. The results of the feedback following the introduction of the pre-printed consent form are shown in Figure 5.

## Discussion

The Montgomery versus Lancashire Health Board case highlighted the importance of the consent process in allowing patients to make the best-informed decision about their procedure [6]. The GMC states that doctors must aim to find what matters most to the patient to best direct the information given to meet these needs [7]. The purpose of the informed consent process is to ensure that the patient is given all the relevant information to make an informed decision, without pressure or duress, about their ongoing management. The DOH and GMC have recommended that the clinician gaining consent for a procedure must be suitably trained and qualified, with sufficient knowledge of the procedure, to ensure any information required by the patient about the procedure may be supplied [2].

The incision and drainage of a superficial skin abscess is a procedure commonly performed within many different settings, ranging from the accident and emergency unit to the operating theatre [5]. The Royal College of Anaesthetics (RCOA) considers incision and drainage of superficial skin abscesses to be suitable for same-day surgery (SDS) [8]. This suitability of SDS has led to the formation of the abscess pathway. The abscess pathway allows haemodynamically stable patients who require an incision and drainage of a superficial skin abscess and who meet a specific criterion to be reviewed and discharged home on the same day of review with the view to receive the operative intervention within 24 hours. The authors felt that by improving the consent process, the abscess pathway could be streamlined, therefore becoming more effective at delivering safe patient care. Standardisation of the consent process can lead to standardised care for the patient. The criteria for the same-day pathway are shown in Table 2.

**Table 2:** Same-day abscess pathway suitability criteria

Suitable for abscess pathway	Not suitable for abscess pathway
Over 18 years old	Younger than 18 years old
1^st^ presentation of abscess in the area	Recurrent abscess in that area
No features of systemic infection	Diabetic/immunosuppressed patient
No features of necrosis	Systemically unwell
Apyrexial	Clinical uncertainty/doubt by the clinician
	Concerns of a deep abscess or a perianal abscess

The inclusion criteria were designed to identify ambulatory patients who would be clinically safe to go home and re-present within 24 hours to have the procedure performed without any compromise to the patient’s health or wellbeing. The exclusion criteria were designed to identify at-risk patients. Those under the age of 18 represent a vulnerable age group and so would require a review by a senior clinician prior to the decision to send them home. Those with recurrent abscesses were deemed to be at risk of having incomplete clearance initially or may have a deeper abscess, so the consensus was that these patients require a senior clinician review prior to a decision about ongoing care. Those who were systemically unwell, diabetic, on immunosuppression, or in whom there was clinical doubt or concern would warrant senior review with or without admission and, therefore, were not suitable for the abscess pathway.

The consent process is described as an opportunity for a suitably trained clinician to have a discussion with a patient, explaining the procedure, risks and benefits, alternative treatments, and the risks and benefits of alternatives [9]. It is important to note that alternative treatment includes no treatment at all [9]. Although leaflets can be used to supplement the consent process, this does not omit the clinician of their responsibility of filling out the above requirements. Consent forms should be used to provide formal consent and written confirmation of this information, allowing the patient to have a copy for their records, thus allowing the opportunity to prompt further questions prior to the procedure. Consent can also be obtained via video consent, and although promising results are noted in studies, the general consensus of the authors is that these should be used to supplement written consent and not replace written consent until further evidence is available to support its use independently [10].

Variations in documentation

The results of this study highlight the variability in consent form documentation. The greatest variability was among the complications documented on consent forms. Incision and drainage of abscesses is a common procedure performed in a variety of settings by a range of clinicians. The risks associated with this procedure are variable, but the common ones, agreed upon by the clinicians in the hospital where this study was performed, include scarring, recurrence, and bleeding. The local population includes a high number of Asian and Afro-Caribbean individuals, so keloid scarring was also deemed vital to be included. Seventeen consent forms (77%) included scarring; however, none specifically referenced keloid scarring.

Six consent forms assessed (27%) did not document whether the procedure was going to be carried out under general or local anaesthesia. General and local anaesthetics have some common risk factors; however, with general anaesthetics carrying a higher degree of risk to the patient, the authors felt that it was vital that this be included [11]. The redesigned pre-printed consent form included a tick box for the clinician to document anaesthetic type, aimed to prompt discussion with the patient about associated risk.

Written information

Providing written information is considered as much a part of the consent process as the act of taking consent. Ensuring the patient has a copy of the consent form can allow the patient to have time to read about the procedure and the risks associated. The study showed that eight (36%) patients had a copy of the consent form given to them. This could have been due to several reasons, including patient refusal; however, this was not documented on the consent form. To combat this issue, a section was added to the pre-printed consent form where clinicians can document whether the patient has refused a copy. Another method to encourage giving a copy to the patient was to print the patient's copy on different coloured paper to act as a memory aid for the clinician.

Legibility

While there is no legal requirement to write in black ink, the Nursing and Midwife Council has stated that documentation should be legible and written in black ink to aid documentation [12]. The results of this study showed that 22 (100%) consent forms were written in black ink. This was attributed to the hospital having supplies of black ink on-site for clinicians to obtain, which would have made compliance with writing in black ink easier. 

The use of pre-printed consent forms will also have the potential to improve legibility and, therefore, patient comprehension. Consent forms can be illegible for a number of reasons, including surgeon time pressures, consent completed in variable settings, and poor handwriting [13]. In this study, 20 consent forms (91%) were noted to be legible. The limiting factor to this was that legibility was from the author's viewpoint. The authors would have worked with the surgeons completing the consent process and, therefore, may have been familiar with the clinician's handwriting, making it easier to recognise lettering and read the consent forms. The use of pre-printed consent forms removes the variability of handwriting, which may limit the patient's understanding of the consent form.

Use of abbreviations

During a discussion with patients about their clinical diagnosis and management, clinicians should aim to maximise understanding by talking slowly and avoiding jargon [14]. The Royal College of General Practitioners (RCGP) carried out research to improve communication with patients. It identified that those from ethnic minority backgrounds, those having low qualifications, those elderly, those without English as a first language, those in the poverty trap, and those with a lower job status struggle more with comprehension of health information [15].

Consent forms provide written information to the patient about their condition and the management options available to them. Thirteen consent forms (59%) used medical abbreviations when discussing the risks of the procedure. The use of medical abbreviations may limit patient understanding, as these terms are not always understood clearly by the patient, which may affect the validity of the consent form [16]. The consensus by the authors was that avoidance of medical abbreviations improves patient understanding, and the development of the pre-printed consent form was done to make the process of consent clearer for both clinician and patient.

Feedback

Following the design of the pre-printed consent form, a talk was given to clinicians within the surgical department, and feedback was sought to assess the usability of the pre-printed consent forms. Out of the clinicians surveyed, 14 out of 14 (100%) found the pre-printed consent forms easy to use and understand, while 12 (86%) would incorporate the pre-printed consent forms within their practice.

Limitations

The study was carried out over a short time period, and although initial feedback from junior doctors was promising, further work and subsequent review of practice, as well as incorporating the consent form into current practice, are required. Furthermore, reauditing following this will establish whether there has been a positive change in the consent process. Further reviews will also highlight any areas of development that may have been overlooked during this cycle, including both the consent process and documentation, allowing the abscess pathway as a whole to improve.

The study did not focus on the grade of an individual carrying out the consent process. Within the NHS hospital where this study was performed, senior house officers and above can consent patients for incision and drainage, but only if they are suitably qualified and have enough knowledge of the procedure to consent in line with GMC and DOH guidelines. Whether there was a link between omissions on the consent form and the grade of the individual carrying out the consent was not assessed and, therefore, is an area for future work.

## Conclusions

The importance of consent cannot be underestimated, especially at a time when medical procedures are constantly improving and patients are more than ever involved in their ongoing care. Patients are seen in a variety of clinical settings, and so the time available is also limited. A method to provide consistent high-quality care can be achieved by standardisation of care, as this will ensure, regardless of time and setting, that the desired outcome of good patient care is achieved.

The use of pre-printed consent forms has great potential to provide relevant procedure-based information to the patient and obtain valid informed consent. The design of a pre-printed consent form for the incision and drainage of the abscess will allow a wide range of suitably trained clinicians to obtain standardised informed consent and so provide a high level of consistent care to all that access the healthcare service.
